# Surgical resection of a ruptured left frontal mycotic aneurysm localized with ICG angiography: a case report

**DOI:** 10.1097/RC9.0000000000000693

**Published:** 2026-07-20

**Authors:** Ning Zhu, Jack Skeggs, Iwan Bennett, Rosalind L. Jeffree

**Affiliations:** a Department of Neurosurgery, The Alfred Hospital, Melbourne, Victoria, Australia; b Department of Infectious Diseases, The Alfred Hospital, Melbourne, Victoria, Australia; c Department of Surgery, Monash University, Clayton, Victoria, Australia

**Keywords:** case report, indocyanine green angiography, infective endocarditis, mycotic aneurysm, surgical resection

## Abstract

**Introduction and importance:**

Ruptured intracranial mycotic aneurysms are rare. There are no established guidelines for management. Limited case reports are available, with no randomized controlled trials.

**Case presentation:**

A 58-year-old male presented with disseminated *Staphylococcus aureus* bacteremia secondary to left lower limb cellulitis. Investigations revealed infective endocarditis and multiple septic emboli in the brain, spleen, and kidney. On day 17 of admission, the patient was found obtunded with global aphasia and right-sided hemiplegia. Computed tomography showed a 57 × 43 × 15 mm left frontoparietal intraparenchymal hemorrhage secondary to a ruptured 6 mm left frontal intracranial mycotic aneurysm. Digital subtraction angiography was not required, and endovascular treatment was unsuitable. The patient underwent craniotomy and resection of the mycotic aneurysm, as well as evacuation of the intraparenchymal hematoma using neuronavigation and intraoperative indocyanine green angiography (ICGA).

**Clinical discussion:**

The aneurysm was difficult to visualize under natural light despite the use of neuronavigation. ICGA provided a very detailed real-time assessment of cerebrovascular architecture and enabled accurate localization and successful resection of the mycotic aneurysm.

**Conclusion:**

ICGA is not commonly used in the surgical resection of intracranial mycotic aneurysms and should be considered if neuronavigation is inadequate for localization.

## Introduction

Intracranial mycotic aneurysms are found in up to 10% of patients with infective endocarditis (IE)[1]. These are mostly caused by bacterial infection. Bacteria from septic emboli infiltrate through the vasa vasorum and spread inwards, causing degradation and weakening of the arterial wall^[^2,3^]^. There are no established guidelines for the management of intracranial mycotic aneurysms[2]. However, unruptured aneurysms are usually treated with intravenous antibiotics for 6+ weeks and serial imaging[3]. Large, enlarging, symptomatic, or ruptured aneurysms warrant endovascular or surgical treatment[1]. This case report has been reported in line with the SCARE checklist[4].

HIGHLIGHTS

- Neuronavigation is recommended in the open surgical resection of mycotic aneurysms.
- Indocyanine green angiography can provide a very detailed, real-time assessment of cerebrovascular architecture and enable accurate localization of intracranial mycotic aneurysms.
- Indocyanine green angiography is not commonly used in surgical resection of mycotic aneurysms and should be considered if neuronavigation is inadequate for localization.

## Case presentation

A 58-year-old male was admitted to a regional hospital after a fall with a prolonged lie. He was confused, in septic shock, and had a large diabetic ulcer over his left ankle with associated cellulitis. His past medical history included type 2 diabetes mellitus and hypertension. He was left-handed and previously independent.

## Investigations

Investigations showed a methicillin-susceptible *Staphylococcus aureus* bacteremia and diabetic ketoacidosis. An insulin/dextrose infusion, intravenous (IV) piperacillin-tazobactam, and IV vancomycin were initiated. Echocardiogram demonstrated IE with a 2 cm mitral valve subvalvular vegetation. A computed tomography (CT) pan scan showed multifocal intracranial infarctions, splenic infarcts, bilateral pyelonephritis, multi-joint septic arthritis, extensive soft tissue thickening, and fat stranding of the left lower leg and foot. A screening CT head angiogram (CTA) was negative for mycotic aneurysms. Magnetic resonance imaging (MRI) demonstrated C5/6 discitis osteomyelitis with anterior epidural phlegmon and a prevertebral abscess extending from C5 to T2.

The patient was transferred to our tertiary hospital on day 5. On arrival, antibiotic therapy was rationalized to Cefazolin. Left midfoot abscesses were thought to be the infective source and were surgically debrided by vascular surgery on day 11. Cardiothoracic surgery planned a vegetectomy on day 12. However, this was cancelled as serial echocardiograms showed resolution of aortic vegetations and reduced mitral subvalvular vegetations.

**Figure 1.**
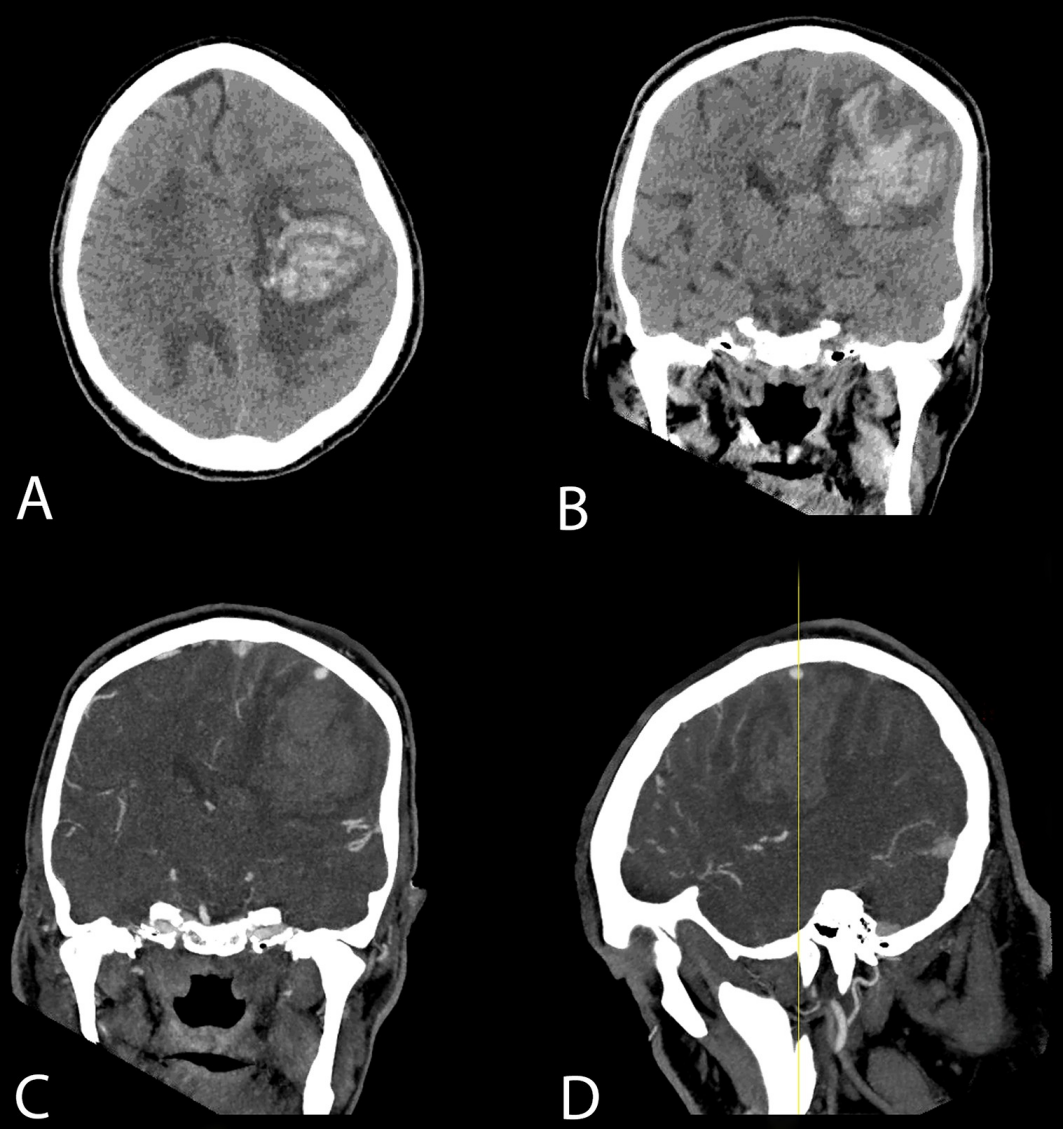
Pre-operative non-contrast CT brain – axial (A), coronal (B) views, and pre-operative CT brain angiogram – coronal (C), sagittal (D) views.

On day 17, the patient became obtunded with global aphasia, right-sided hemiplegia, and neglect. CT revealed a 57 × 43 × 15 mm left frontoparietal intraparenchymal hemorrhage with intraventricular extension into the lateral and third ventricles. CTA showed a new 6 mm focal rounded enhancing focus at the cortex overlying the hemorrhage, suggestive of a mycotic aneurysm (see Fig. 1). Interventional neuroradiology deemed the CTA adequate for diagnosis without digital subtraction angiography (DSA) and agreed with the finding of a mycotic aneurysm.

## Treatment

Endovascular treatment was unsuitable due to the aneurysm’s distal location and the need for the sacrifice of a parent vessel also supplying the motor cortex.

Subsequently, the patient underwent a craniotomy and resection of the left frontal mycotic aneurysm with the primary intention of preventing re-rupture. The intraparenchymal hematoma was also evacuated.

**Figure 2.**
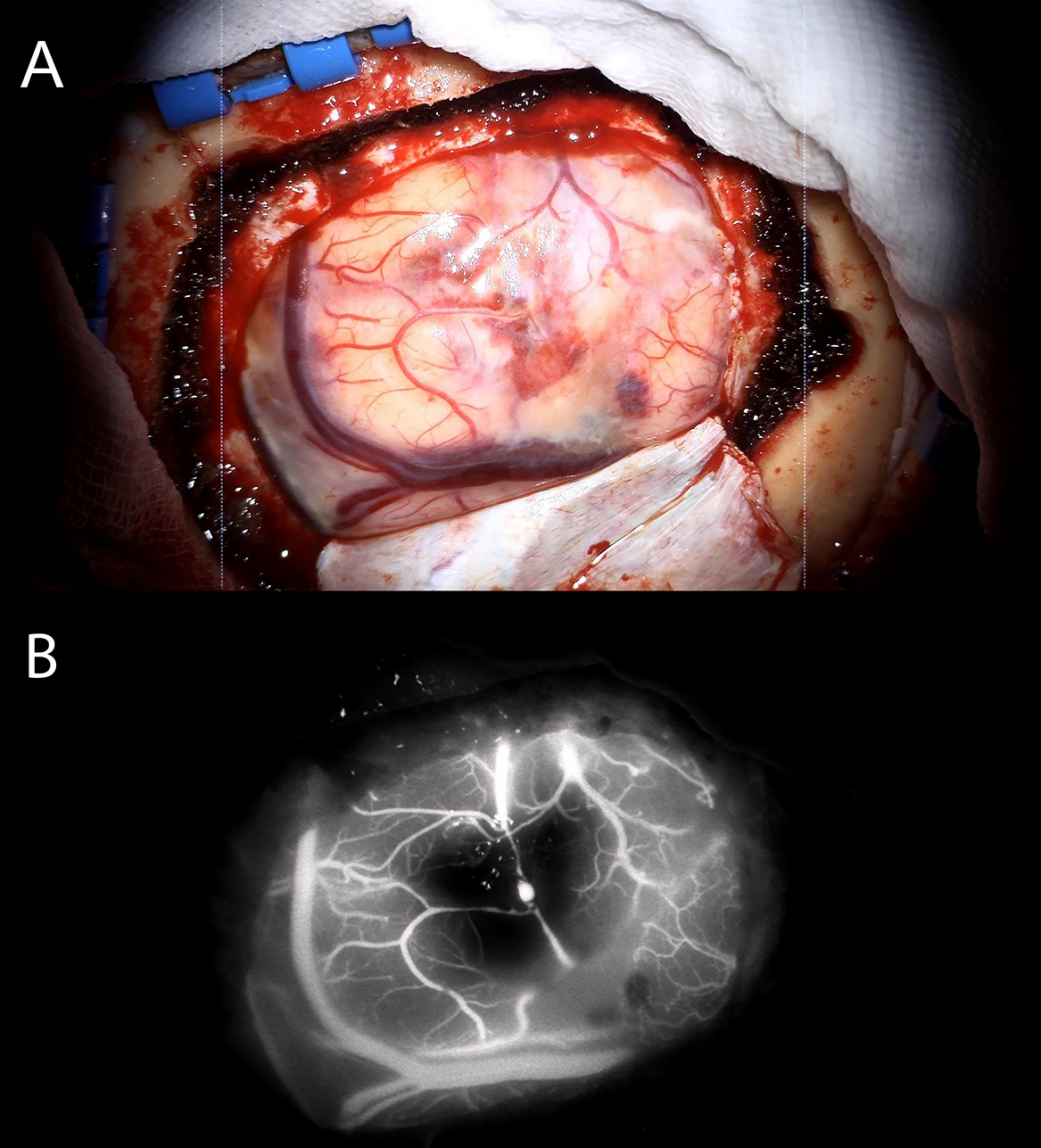
Intraoperative photo of the surgical field (A) and intraoperative indocyanine green angiography (ICGA) (B).

Neuronavigation (Medtronic Stealth) was used to plan the craniotomy and approximately locate the mycotic aneurysm. The craniotomy revealed a tight, swollen brain, abnormal vasculature on the brain surface, and small areas of purulent material in adjacent gyri (see Fig. 2A). In the cortical area indicated by neuronavigation, the mycotic aneurysm was not easily visualized or identifiable under natural light.

Intraoperative indocyanine green angiography (ICGA) was utilized to characterize the cerebrovascular architecture and identify the mycotic aneurysm (see Fig. 2B). Three points of arterial connection were observed and were successfully disconnected with bipolar diathermy 5 mm distal to the aneurysm. These points were joined to form a 5 mm circumference of cortex around the aneurysm, which was resected *en bloc* and sent for histological analysis. This provided access to the underlying hematoma, which was evacuated in segments using forceps and suction. Tissue culture confirmed *Staphylococcus aureus*, while histopathology revealed acutely inflamed and necrotic dilated blood vessels with vessel walls diffusely infiltrated by acute inflammatory cells (see Supplemental Digital Content Figure 1, available at: https://links.lww.com/IJSCR/A96). IV Linezolid was initiated in addition to Cefazolin to ensure adequate cerebral penetration, given the extensive burden of infection.

## Outcome

**Figure 3.**
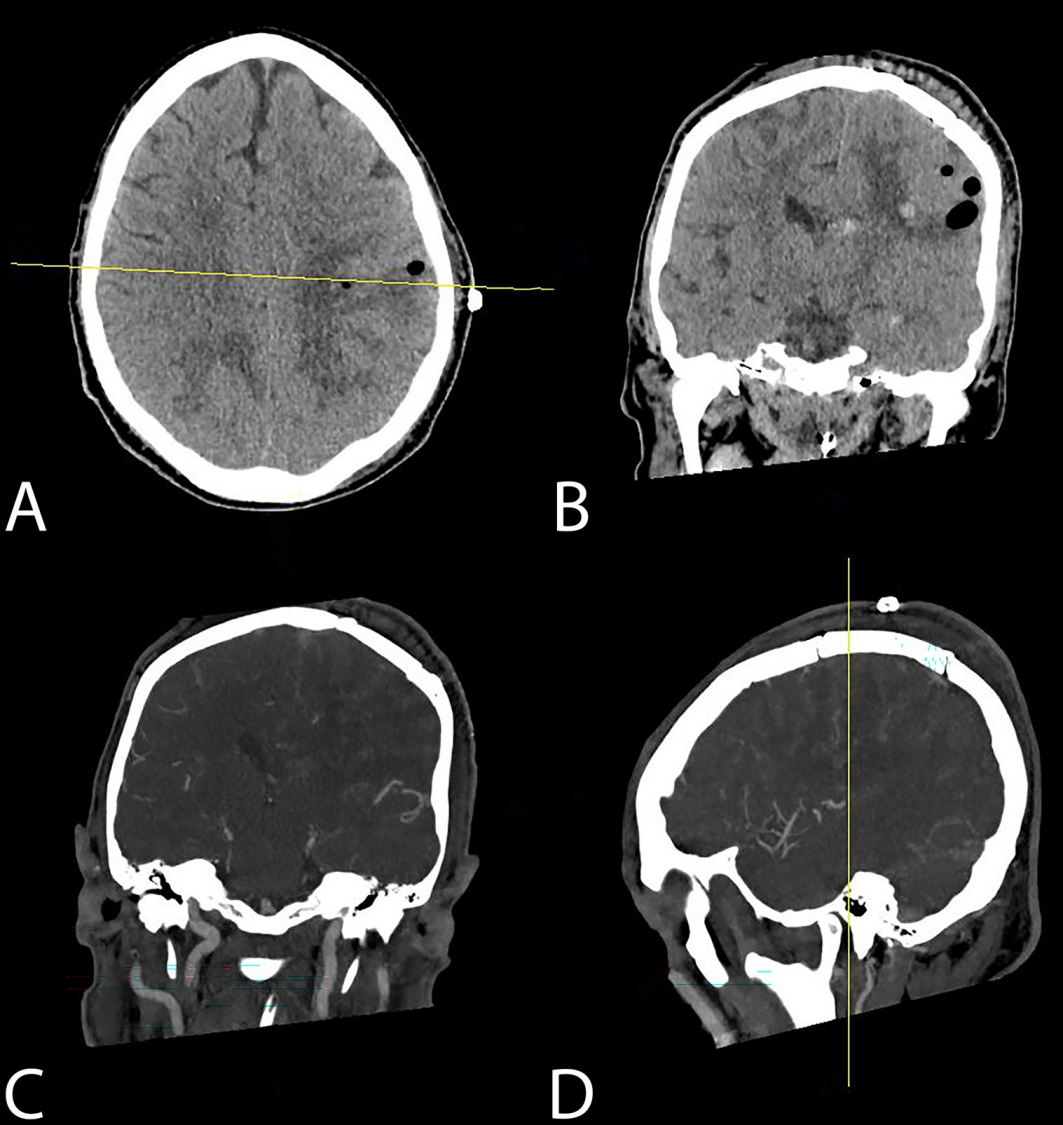
Post-operative non-contrast CT brain – axial (A), coronal (B) views, and post-operative CT brain angiogram – coronal (C), sagittal (D) views.

Post-operatively, the patient was confused, with ongoing functional communication deficits and ongoing right-sided hemiplegia. CTA did not show any residual aneurysm or new aneurysms (see Fig. 3).

The patient developed biopsy-confirmed acute interstitial nephritis secondary to IV Cefazolin and was transitioned to IV Clindamycin on day 39. This was stepped down to oral Clindamycin. Twelve weeks of total antibiotics were given following major source control due to the presence of an undrained epidural abscess. Given the need for prolonged antibiotic therapy, Clindamycin was chosen over Linezolid to avoid toxicity.

The patient was transferred back to the referring regional hospital for ongoing acute care and rehabilitation on day 50. Clinical assessment at 9 months demonstrated improved right-sided neurology with 3/5 power in the upper and lower limbs.

## Discussion

Mycotic aneurysms are localized vascular dilatations secondary to infectious degradation of the arterial wall[5]. Intracranial mycotic aneurysms are found in 2–10% of patients with IE[1]. In all, 72.8% are caused by bacterial infection, 13.2% by fungal infection, and the remainder by viral or mycobacterial infection. *Streptococcus viridans* and *Staphylococcus aureus* are the most common causative organisms^[^2,6^]^. Our patient’s mycotic aneurysm was caused by a disseminated *Staphylococcus aureus* bacteremia.

Mycotic aneurysms often develop at arterial branch points and are most common in the anterior circulation (50–78%), particularly the middle cerebral artery[2]. They are usually multiple and found on distal branches[7]. Our patient had a single aneurysm, which was located distally and in the anterior circulation. The risk of rupture is <2%[2]. The mortality increases from 30% to 80% following rupture[8].

The latest guidelines for the management of IE do not recommend routine vascular imaging unless neurological complications are suspected[9]. Our patient underwent a screening CTA on day 4; however, this was negative, likely due to the early timing in the patient’s disease course. The time between IE diagnosis and rupture of a mycotic aneurysm ranges from 2 to 5 weeks[1]. Our patient’s aneurysm ruptured on day 17.

DSA was previously the gold standard for the diagnosis of intracranial aneurysms[9]. However, the development of multi-detector CT has allowed for an increase in resolution and complete visualization of the vascular tree with a lower contrast burden and risk of permanent neurological deficit than DSA. CTA has a relative sensitivity of 90% and specificity of 86%[3]. In our case, the interventional neuroradiologists deemed the CTA to be of sufficient quality and concluded that a DSA was not required.

There are no established guidelines for the management of mycotic aneurysms[2], with only a limited number of case reports and no randomized controlled trials^[^3,10^]^. Unruptured mycotic aneurysms are usually treated with intravenous antibiotics for at least 6 weeks, with serial angiography to monitor aneurysmal size and demonstrate resolution[3].

In the absence of increased intracranial pressure, mass effect, hypotension, hematoma, or involvement of eloquent territory, endovascular therapy has been favored. Endovascular techniques include parent artery occlusion, coil embolization, flow diversion, or liquid embolization agents[11] and have a lower rate of mortality and anesthetic complications, rapid institution of anticoagulation therapy, and a shorter delay to cardiac surgery[11]. Multiple or surgically inaccessible aneurysms should be treated with endovascular therapy[12]. The distal and eloquent location in our case prevented endovascular treatment.

For large, enlarging, symptomatic, or ruptured mycotic aneurysms, endovascular or surgical treatment is indicated[1]. Ruptured aneurysms associated with a large intraparenchymal hematoma generally require open surgery[3]. Open surgical techniques include aneurysm clipping, aneurysm resection, and parent artery trapping/occlusion with or without bypass[12]. Our patient proceeded to surgery with the primary intention of resecting the aneurysm to prevent further hemorrhage. Evacuation of the intraparenchymal hemorrhage was performed as a secondary goal. The patient was clinically stable, and relief of mass effect would not improve the patient’s functional neurology.

The use of neuronavigation has been recommended, as most failed surgical attempts have been found to be secondary to a failure to localize the mycotic aneurysm intraoperatively, despite it being visualized on pre-operative angiography[12]. In our operation, the mycotic aneurysm was difficult to identify among the abnormal vasculature on the surface of the brain under natural light (see Fig. 2A). Neuronavigation allowed for the identification of the general location of the mycotic aneurysm but was inadequate.

ICGA is usually used to confirm complete occlusion or obliteration of non-infectious aneurysms and facilitate intraoperative clip adjustment if necessary[13]. It has recently been shown to be an important tool for the surgical treatment of mycotic aneurysms, but its use is not common^[^13,14^]^. In our case, intraoperative ICGA proved to be invaluable as it allowed for very detailed real-time imaging of the cerebrovascular architecture and accurate localization of the mycotic aneurysm (see Fig. 2B). The arterial supply to the aneurysm was easily identified, which facilitated intraoperative decision-making to enable a successful resection.

A strength of this case was the multidisciplinary management of the patient by multiple medical and surgical teams. A weakness of this study was that it was only a single case, and our findings cannot be used to establish generalized therapeutic effectiveness. Further research will be required to investigate whether ICGA should be used as a routine intraoperative investigation in the open surgical resection of mycotic aneurysms.

## Conclusion

In open surgical resection of a ruptured mycotic aneurysm, the use of neuronavigation is recommended to localize the aneurysm. In our case, the aneurysm was difficult to identify under natural light despite using neuronavigation. ICGA provided detailed real-time assessment of the patient’s cerebrovascular architecture and enabled accurate localization and successful resection of the mycotic aneurysm. ICGA is not commonly used in the surgical resection of mycotic aneurysms and should be considered if neuronavigation is inadequate for localization.

## Data Availability

The data that support the findings of this study are available from the corresponding author, N.Z., upon reasonable request.

## Notes

N.Z.: Data curation, formal analysis, investigation, methodology, writing, review, and editing. J.S.: Review and editing. I.B.: Supervision, conceptualization, review, and editing. L.J.: Supervision, conceptualization, review, and editing.

## Research registration unique identifying number (UIN)

The author confirms that research registration was not applicable.
